# A draft genome for the African crocodilian trypanosome *Trypanosoma grayi*

**DOI:** 10.1038/sdata.2014.24

**Published:** 2014-08-05

**Authors:** Steven Kelly, Alasdair Ivens, Paul T. Manna, Wendy Gibson, Mark C. Field

**Affiliations:** 1 Department of Plant Sciences, University of Oxford, Oxford OX1 3RB, UK; 2 Centre for Immunity, Infection and Evolution, Ashworth Laboratories, University of Edinburgh, Edinburgh EH9 3JT, UK; 3 Division of Biological Chemistry and Drug Discovery, University of Dundee, Dundee DD1 5EH, Scotland, UK; 4 School of Biological Sciences, University of Bristol, Bristol BS8 1UG, UK

## Abstract

The availability of genome sequence data has greatly enhanced our understanding of the adaptations of trypanosomatid parasites to their respective host environments. However, these studies remain somewhat restricted by modest taxon sampling, generally due to focus on the most important pathogens of humans. To address this problem, at least in part, we are releasing a draft genome sequence for the African crocodilian trypanosome, *Trypanosoma grayi* ANR4. This dataset comprises genomic DNA sequences assembled *de novo* into contigs, encompassing over 10,000 annotated putative open reading frames and predicted protein products. Using phylogenomic approaches we demonstrate that *T. grayi* is more closely related to *Trypanosoma cruzi* than it is to the African trypanosomes *T. brucei*, *T. congolense* and *T. vivax*, despite the fact *T. grayi* and the African trypanosomes are each transmitted by tsetse flies. The data are deposited in publicly accessible repositories where we hope they will prove useful to the community in evolutionary studies of the trypanosomatids.

## Background & summary

Most trypanosomatid parasites of humans, livestock and plants are transmitted between hosts by invertebrate vectors. They are widespread, and are collectively responsible for substantial economic and health losses in many of the world’s poorest regions. Within this group are the *Leishmania* species, the causative agents of Leishmaniasis, as well as the monophyletic *Trypanosoma* genus, which includes *Trypanosoma cruzi* and *Trypanosoma brucei*, causative organisms of Chagas disease and African trypanosomiasis respectively. Despite a probable single origin of vertebrate parasitism within this monophyletic group^1,2^, the challenge of escaping clearance by host immune responses has given rise to a variety of disparate parasitic lifestyles including intracellular parasitism (*Leishmania spp.* and *T. cruzi*) and antigenic variation (*T. brucei*)^3–5,3–5,3–5^.

Since the publication of the *T. cruzi*, *T. brucei* and *Leishmania major* genomes in 2005^6–8,6–8,6–8^, several other trypanosomatid genome sequences have been released, including further *Leishmania* species and other African tsetse transmitted trypanosomes related to *T. brucei* ^9–12,9–12,9–12,9–12^. The availability of sequence data for other *T. brucei* clade trypanosomes has increased our understanding of the evolution of the primary immune evasion strategy of this parasite as well as the evolution of cell surface molecules that represent the host-parasite interface^12,13^. Similarly, sequencing of disparate *T. cruzi* isolates has provided major insights into population structure and dynamics^14,15^. Though more species now have published genome sequences, sampling across the trypanosomatid phylogeny is limited and thus there are limited resources for comparative genomic investigations.

To address this key knowledge gap, here we provide a draft genome sequence of the African trypanosomatid parasite of crocodiles, *Trypanosoma grayi* (Data citation 1 and Data Citation 2). *T. grayi* is an extracellular parasite of the bloodstream of crocodiles, and though it is transmitted by tsetse flies it is closely related to other trypanosome parasites of crocodiles in South America^16^. The trypanosome is taken up by tsetse flies in a bloodmeal and resides solely within the mid- and hindgut. Unlike salivarian trypanosomes, transmission between crocodile hosts occurs *via* oral contamination with infective metacyclics in tsetse faeces^17,18^. This faecal transmission strategy is employed by many other trypanosomes, including *T. cruzi*.

**Figure 1:**
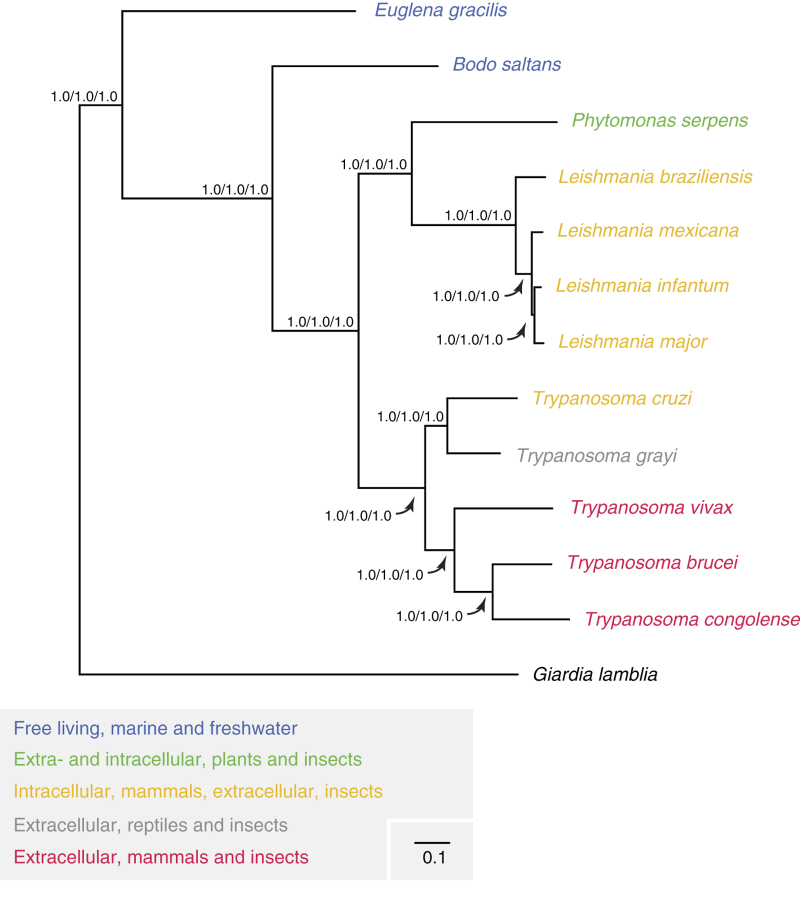
Phylogenetic tree of selected Euglenozoa. Phylogeny is inferred from 119,006 aligned amino acid positions (1,547,078 amino acids) from 959 nuclear genes. The topology was calculated using Bayesian inference (BI), bootstrapped maximum likelihood (ML) analysis and bootstrapped neighbor joining (NJ). ML used the JTT model of amino acid substitution and CAT rates, BI used the WAG model of amino acid substitution and gamma distributed rates approximated by four discrete gamma categories. Branch lengths shown are from the ML topology, scale bar indicates number of changes per site. Values shown at internal nodes represent bootstrap support values for ML and NJ tree as well as posterior probabilities for BI tree. In all cases support for each bipartition in the topology is 100%. *Giardia lamblia*, a diplomonad excavate, is included as an outgroup as the Euglenozoa are also members of the Excavata.

BLAST and OrthoMCL analysis of the genome sequence and predicted gene models respectively suggests that *T. grayi* possesses neither the *T. brucei* type VSG surface antigens nor the *T. cruzi* type mucin coat. Thus *T. grayi* may have evolved an alternative family of primary surface antigen genes, or possess a novel immune evasion strategy geared to survival in the reptilian bloodstream^19^. Both phylogenomic reconstruction and best-BLASTp analysis demonstrate that *T. grayi* is more closely related to *T. cruzi* than to *T. brucei* (Figure 1 and Table 1). This result refines the phylogenetic position of *T. grayi,* that in previous studies using 18S ribosomal RNA and glycosomal glyceraldehyde dehydrogense (gGAPDH) genes was placed in a separate clade from both *T. cruzi* and *T. brucei*, often with other reptile or bird trypanosomes^20–22,20–22,20–22^. Additional taxon sampling in this region of the phylogenetic tree will be important for resolving these relationships further. We anticipate that these data will provide a useful comparator for evolutionary studies of the adaptations of trypanosomes to different vertebrate hosts, as well as increasing the available sequence data resources for this globally important group of parasites.

To generate the draft genome, DNA from *T. grayi* strain ANR4, isolated from the midgut of the tsetse fly *Glossina palpalis gambiensis* in The Gambia^20^ was sequenced by 91 bp paired-end Illumina sequencing and assembled *de novo* into contigs (Data citation 2). We inferred the phylogenetic position of *T. grayi* strain ANR4 through construction of a concatenated protein sequence phylogeny using 959 single copy nuclear encoded genes. We also confirmed that both the 18S ribosomal RNA sequence and gGAPDH sequence for our *T. grayi* strain ANR4 were 100% identical to those provided in GenBank (AJ005278 and AJ620257 respectively) for *T. grayi*. Furthermore, we have identified and annotated over 10,000 putative open reading frames and have submitted this information to public databases alongside the draft genome sequence.

## Methods

### Sequencing and assembly

**Figure 2:**
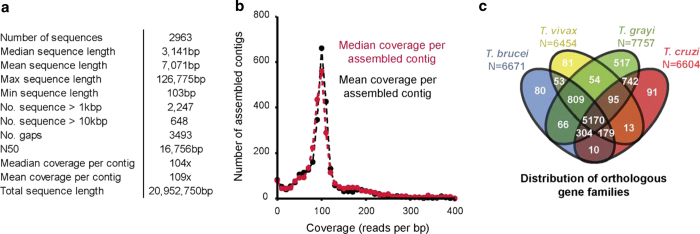
Assembly statistics. (**a**) General assembly statistics describing assembled contig length and coverage. (**b**) Graph showing distribution of coverage estimates for each assembled contig estimated using median and mean coverage depth. (**c**) Venn diagram showing the distribution of orthologous gene families in four of the species used for OrthoMCL clustering.

*T. grayi* strain ANR4 was grown *in vitro* in Cunningham’s medium and genomic DNA was extracted from agarose plugs using standard phenol/chloroform methods. DNA was sequenced by 91 bp paired-end Illumina sequencing at the Beijing Genomics institute (www.genomics.cn/en/). Raw reads were subject to quality filtering using trimmomatic^23^. This was done to remove low quality bases and read-pairs as well as contaminating adaptor sequences prior to assembly. Sequences were searched for all common Illumina adaptors (the default option) and the settings used for read processing by trimmomatic were ‘LEADING:10 TRAILING:10 SLIDINGWINDOW:5:15 MINLEN:50’. The quality filtered paired-end reads were then subject to read error correction using the ALLPATHS-LG^24^ ErrorCorreactReads.pl program using the default program settings. The corrected reads were then assembled using SGA^25^ using default settings and setting the mimimum overlap length to 80. The assembled contigs were scaffolded by mapping the trimmed and filtered paired-end reads (described above) to the assembled contigs using BWA-MEM and scaffolding the contigs using the SGA^25^ scaffolding algorithm using default program settings. The resultant scaffolds were then subject to fourteen rounds of assembly error correction and gap filling using Pilon (http://www.broadinstitute.org/software/pilon/) using the ‘–fix all’ option and setting the expected ploidy to diploid. Following scaffolding and assembly error correction all filtered paired-end reads were mapped to the contig set using BWA-MEM^26^, paired-end reads that did not map to the assembly were isolated and the above assembly, scaffolding and correction process was repeated until all no-further reads could be assembled. The final draft assembly contained 2,963 sequences greater than 100 bp in length with an N50 of 16.7 kb and a total assembly length of 20.9 Mb and average coverage per assembled contig of ~105X (Figure 2a,b).

### ORF finding and annotation

The assembled draft genome of *T. grayi* was subject to gene model prediction using Augustus^27^. In brief, an initial set of gene models were predicted using gene prediction parameters inferred by training Augustus using the set of genes currently annotated in the *T. cruzi* genome. These gene model parameters were used to predict a training set of genes in the draft assembly of *T. grayi*. The training set of genes were then used for multiple iterations of prediction and training until prediction converged on a final set of gene models and no further genes could be detected. The identity of the *T. grayi* DNA used for sequencing was confirmed against database sequences for 18S ribosomal RNA and glycosomal GAPDH genes (AJ005278 and AJ620257 respectively).

### Gene family analysis

The protein sequence files for a subset of available trypanosomatid genomes were downloaded from TriTrypDB. These were combined with the newly predicted protein sequences from *T. grayi* and subject to orthologue group clustering using OrthoMCL^28^. The presence of gene families in each species was analyzed and the overlap in gene family content between each species and that of the newly assembled *T. grayi* genome was compared. On average the predicted gene model set of *T. grayi* contained 95% of the gene families present in *T. brucei, T. vivax* and *T. cruzi* (Figure 2c). To put this in context, *T. cruzi* and *T. vivax* contain 84 and 93% of the gene families present in *T. brucei* respectively (Figure 2c).

### Phylogenetics for strain verification

Orthologous sequence groups that contained only single copy genes in each of the species that were subject to clustering were selected (*n*=959). These single copy gene families were aligned using MergeAlign^29^ and concatenated to form a super-alignment containing 119,006 aligned amino acid positions across all species (1,547,078 amino acids). This concatenated alignment was subject to phylogenetic inference using bootstrapped maximum likelihood, Bayesian inference and bootstrapped neighbor joining methods. Maximum likelihood trees were inferred using FastTree^30^, utilizing the JTT model of amino acid substitution and CAT rates. A Bayesian inference tree was inferred using MrBayes v3.1.2^31^ using the WAG model of amino acid substitution and gamma distributed rates approximated by four discrete gamma categories. Two runs each of four chains were initiated and allowed to run for 200,000 generations sampling every 500 generations. Convergence was assessed through visual inspection of log-likelihood traces and through analysis of the standard deviation of split frequencies. The analysis had reached stationary phase after 15,000 generations and these first 15,000 generations were discarded as burnin prior to inferring the consensus tree. The neighbor joining tree was inferred using QuickTree^32^ using the default parameters. The final topology is shown in Fig. 1 and received 100% support at each bipartition from all methods.

## Data Records

Data are available both via GenBank as (accession numbers JMRU01000001 to JMRU01002871) and as contigs (accession JMRU00000000.1) under BioProject PRJNA244495, BioSample SAMN02726834 (Data Citation 1). Raw read files are at NCBI SRA under experiment accession SRX620256 and run accession SRR1448313 (Data Citation 2).

Data are also available at TriTrypDB^33^ as a hosted genome integrated with other trypanosomatid datasets, http://tritrypdb.org/tritrypdb/showApplication.do (search for all annotated genes), http://tritrypdb.org/common/downloads/Current_Release/TgrayiANR4/ (file download) and http://tritrypdb.org/tritrypdb/getDataset.do?datasets=tgraANR4_primary_genome_RSRC for dataset description.

## Technical Validation

**Figure 3:**
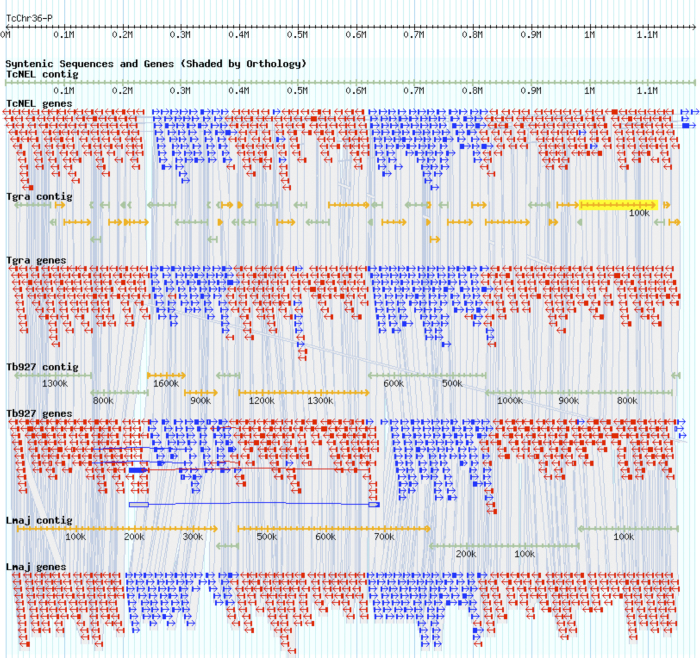
Alignment of ***T. grayi*** contigs against syntenic region of ***T. cruzi*** (Chr 36), ***T. brucei*** and ***L. major***. *T. grayi* assembled contigs were mapped to a contig from Chr 36 of *T. cruzi* (TcNEL, top) in Artemis (http://www.sanger.ac.uk/resources/software/artemis/) together with the equivalent syntenic region from *L. major*. Transcripts (red, blue) are shown beneath mapped contigs (teal, orange) and orthologous sequences are shown as lines behind the main graphic. Despite the fragmentary nature of the *T. grayi* dataset, the data map well to this highly conserved region of the trypanosomatid genomes. Tb927 is the *Trypanosoma brucei* TREU927 genome strain. TcNEL is the *Trypanosoma cruzi* CL Brenner non-Esmereldo-like genome strain.

The contig statistics of the assembly are reported in Figure 2, and an example region of an assembly against several related trypanosomatid genomes is shown in Figure 3. Phylogenetic strain validation as described above confirmed the placement of *T. grayi* ANR4 with other species of genus *Trypanosoma* (Fig. 2) and identity of the sequenced genome here with the previously reported 18S and glycosomal GAPDH genes (AJ005278 and AJ620257 respectively). The phylogenomic position of *T. grayi* closer to *T. cruzi* than *T. brucei* is also supported by BLASTp analysis of all predicted open reading frames (Table 1).

## Additional information

**How to cite this article:** Kelly, S. *et al.* A draft genome for the African crocodilian trypanosome *Trypanosoma grayi*. *Sci. Data* 1:140024 doi: 10.1038/sdata.2014.24 (2014).

## Supplementary Material



## Figures and Tables

**Table 1:** BLASTp similarity scores for *T. grayi* predicted proteins with a bitscore value of >75 using the full non-redundant database from NCBI.

**Number of predicted genes**	**Species**
3,709	*Trypanosoma cruzi* strain CL Brener
2,109	*Trypanosoma cruzi* marinkellei
1,113	*Trypanosoma cruzi*
694	*Trypanosoma cruzi* Dm28c
194	*Trypanosoma vivax* Y486
164	*Trypanosoma brucei brucei* strain 927/4 GUTat10.1
156	*Trypanosoma brucei gambiense* DAL972
132	*Trypanosoma congolense* IL3000
44	*Angomonas deanei*
36	*Trypanosoma rangeli*
27	*Strigomonas culicis*
26	*Trypanosoma brucei* TREU927
22	*Leishmania major* strain Friedlin
17	*Leishmania braziliensis* MHOM/BR/75/M2904
17	*Leishmania infantum* JPCM5
16	*Leishmania guyanensis*
Number of ‘top hits’ with a bitscore value of >75 for each trypanosomatid species are reported. Database was interrogated on 28 February 2014, using BLAST 2.2.27+.	
